# New parasites and predators follow the introduction of two fish species to a subarctic lake: implications for food-web structure and functioning

**DOI:** 10.1007/s00442-012-2461-2

**Published:** 2012-09-28

**Authors:** Per-Arne Amundsen, Kevin D. Lafferty, Rune Knudsen, Raul Primicerio, Roar Kristoffersen, Anders Klemetsen, Armand M. Kuris

**Affiliations:** 1 Department of Arctic and Marine Biology, Faculty of Biosciences, Fisheries and Economics, University of Tromsø, 9037 Tromso, Norway; 2 U.S. Geological Survey, Western Ecological Research Center, c/o Marine Science Institute, UC Santa Barbara, Santa Barbara, CA 93106 USA; 3 Department of Ecology, Evolution and Marine Biology, and Marine Science Institute, University of California, Santa Barbara, CA 93106 USA

**Keywords:** Non-native species, Pelagic community, Species additions, Topology, Trophic interactions

## Abstract

Introduced species can alter the topology of food webs. For instance, an introduction can aid the arrival of free-living consumers using the new species as a resource, while new parasites may also arrive with the introduced species. Food-web responses to species additions can thus be far more complex than anticipated. In a subarctic pelagic food web with free-living and parasitic species, two fish species (arctic charr *Salvelinus alpinus* and three-spined stickleback *Gasterosteus aculeatus*) have known histories as deliberate introductions. The effects of these introductions on the food web were explored by comparing the current pelagic web with a heuristic reconstruction of the pre-introduction web. Extinctions caused by these introductions could not be evaluated by this approach. The introduced fish species have become important hubs in the trophic network, interacting with numerous parasites, predators and prey. In particular, five parasite species and four predatory bird species depend on the two introduced species as obligate trophic resources in the pelagic web and could therefore not have been present in the pre-introduction network. The presence of the two introduced fish species and the arrival of their associated parasites and predators increased biodiversity, mean trophic level, linkage density, and nestedness; altering both the network structure and functioning of the pelagic web. Parasites, in particular trophically transmitted species, had a prominent role in the network alterations that followed the introductions.

**Electronic supplementary material:**

The online version of this article (doi:10.1007/s00442-012-2461-2) contains supplementary material, which is available to authorized users.

## Introduction

Food webs depict ecological communities via networks of trophic relationships, and the structure and complexity of these networks influence community dynamics and stability (Bascompte et al. 2003; Dunne et al. 2005; Allesina and Pascual 2008). Beyond their importance in addressing general ecological questions, food-web analyses of species additions and deletions can also shed light on the impact of invasions and extinctions (Dunne et al. 2002; Srinivasan et al. 2007; Petchey et al. 2008). In particular, species introductions, in addition to increasing species richness, can alter food-web topology because a new species can act as a consumer or resource for existing species, or might be the critical resource needed for other consumers to invade the web. Introduced or invading species are furthermore likely to have hitchhiking parasites (Prenter et al. 2004; MacLeod et al. 2010), and parasites may also be affected by additions or extirpations of their hosts (Hechinger and Lafferty 2005), which can both affect food-web structure and functioning.

Most free-living species have several parasite species (Poulin and Morand 2004; Hudson et al. 2006). Although not often incorporated into food webs (Marcogliese and Cone 1997; Byers 2009), these infectious agents may affect network structure (Lafferty et al. 2006, 2008; Hernandez and Sukhedo 2008; Amundsen et al. 2009). Parasites are also likely to increase food-web complexity (Hudson et al. 2006) and alter ecosystem stability (Dobson et al. 2006; Wood et al. 2007). Inclusion of parasites in food webs can therefore change the way we view communities and ecosystem dynamics (Marcogliese and Cone 1997; Lafferty et al. 2008; Byers 2009; Poulin 2010), also including the consequences of introduced species (Torchin et al. 2003, 2005; Prenter et al. 2004; Thieltges et al. 2009). For instance, the replacement of a native snail by an introduced species (Torchin et al. 2005) had no effect on food-web topology unless one considered parasites (Lafferty and Kuris 2009); the introduced snail had a single parasite species and its replacement of the native species resulted in the extirpation of more than a dozen native parasite species. Alternatively, if introduced species do not extirpate native hosts, the diversity of parasites in a system could increase. For example, Torchin et al. (2003) estimated that, for every introduced free-living species, two parasite species on average are also introduced. As a result, an increase in the diversity of free-living species may also increase the diversity of parasitic species (Hechinger and Lafferty 2005).

Pelagic systems (both lacustrine and marine) have been used as model food webs (e.g. Havens 1997; Matveev 2003; Jonsson et al. 2005). Here, we evaluate how the introduction of two fish species altered the pelagic food web of a subarctic lake. Although the lake is a coupled benthic–pelagic system, our results pertain only to the pelagia. Two abundant fish species have a known history of deliberate introductions to Lake Takvatn, northern Norway (Jørgensen and Klemetsen 1995; Amundsen et al. 2007). Because they were introduced from nearby, some of their parasites and predators could have accompanied or followed them. Unfortunately, there were no observations of the food web before the introductions. Therefore, we used a conservative heuristic reconstruction of the pre-introduction web (the results were not sensitive to alternative reconstructions; see Online Resource). This retrospective analysis constitutes a practical implementation of the species addition/deletion approach that has been used to explore network impacts of invasions and extinctions (Srinivasan et al. 2007; Petchey et al. 2008). The analysis suggests that the fish introductions have led to new trophic links related to the arrival of parasites and predatory birds in the pelagic network. Hence, we hypothesised that the fish introductions affected the topology of the pelagic web, leading to distinct alterations of important food-web characteristics, including changes in the distribution of body sizes of species in the system. We further hypothesised that parasites have an important role for the structural and dynamic implications of these species additions.

## Materials and methods

### The study system and current food web

The study lake, Takvatn (69°07′N, 19°05′E), is a subarctic, oligotrophic and dimictic lake situated 214 m above sea level in northern Norway with an area of 14.2 km^2^ and a maximum depth of ca 80 m. Mountains and a landscape dominated by birch (*Betula pubescens*) with scattered pine trees (*Pinus* *sylvestris*) and patches of farmland surround the watershed. The climate is subarctic with an average air temperature in July of 13.2 °C. Midnight sun is present from late May until late July, whereas the polar night lasts from late November to late January. The ice-free season extends from June to November, with summer stratification from mid-July to the end of August. The maximum epilimnetic temperature is approximately 14 °C, and the thermocline occurs at 10–14 m depth. Secchi depth ranges between 14 and 17 m, and total phosphorus concentration does not exceed 5 μg l^−1^. The pH is neutral to slightly alkaline.

Detailed information related to phytoplankton, zooplankton, fish, birds and parasites and their trophic interactions are available from long-term ecological studies in Takvatn over the last three decades (e.g. Primicerio 2000; Amundsen et al. 2007, 2009; Persson et al. 2007). These data were used to develop a comprehensive topological food web for the pelagia of the lake (hereafter called the post-introduction food web; see Amundsen et al. 2009 for details).

The established food web is a source web, including only trophic relationships among species that arise from phytoplankton as the basal energy source. To best represent the available data, the food web was constrained to species using the open-water limnetic zone as their feeding habitat during the ice-free season of the lake (see also Havens 1997 for an extensive treatise of pelagic food webs). This is the period when the food web is most diverse, but it also means that our results do not apply to the winter food web (which, for instance, does not include birds). Winter dynamics are different in that there is lower diversity, productivity, and metabolism. With respect to parasites, only taxa completing their life cycle by using pelagic-dwelling species as their hosts have been included as members of the pelagic network.

### Estimating the web before fish introduction

Originally, brown trout *Salmo trutta* was the only fish species present in Takvatn, but after overexploitation of the trout population, arctic charr *Salvelinus alpinus* were deliberately introduced from the nearby lake Fjellfrøsvatn in 1930 (Amundsen et al. 2007). Takvatn and Fjellfrøsvatn are located 6 km apart in different tributaries of the same watershed. Three-spined stickleback *Gasterosteus aculeatus* were introduced to Takvatn in 1950 from Sagelvvatn, another nearby lake located 13 km away in a different watershed (Jørgensen and Klemetsen 1995). This was a deliberate introduction to improve forage for brown trout and arctic charr by adding a prey fish species. No direct connections exist between the three lake systems, and the aquatic communities of these postglacial lakes have been isolated for several thousand years. To our knowledge, both introductions included adults which were not inspected for the presence of parasites. Based on the knowledge of the established food web and the known history of the introductions of arctic charr and three-spined stickleback, we heuristically reconstructed the pelagic food web in Takvatn prior to the introduction of the fishes (hereafter called the pre-introduction food web). First, we removed arctic charr and three-spined stickleback and their associated links from the list of nodes in the current pelagic web. Thereafter, we omitted from the food web (1) parasite species that require arctic charr and/or three-spined stickleback to complete their life cycle, and (2) predator species that had no pelagic prey other than arctic charr and/or three-spined stickleback.

Our heuristic reconstruction of the pre-introduction network follows a conservative approach, only omitting species that rely upon arctic charr and/or three-spined stickleback as obligate nutritional resources and thus evidently could not have been present in the pelagic web prior to the arrival of these introduced species. Lacking from our reconstructed web are any species driven extinct by the introduced fishes. Top–down predation effects may have occurred following the fish introductions with potential consequences for the species composition at lower trophic levels. Arctic charr and three-spined stickleback are efficient zooplankton predators (e.g. Langeland and Nøst 1995), and their arrival may thus have negatively influenced predation-vulnerable species, resulting in changes in the composition of the zooplankton community. Similarly, the phytoplankton composition of the pre-introduction web may also have differed from the post-introduction web due to subsequent trophic cascade effects. Although no information is available to corroborate extinctions, we did consider plausible alternative webs where the fish introductions led to, e.g., the extinction of a large-bodied cladoceran as well as other potential scenarios for changes in the zooplankton and phytoplankton assemblages (see Online Resource). These alternative scenarios revealed only minor differences in important food-web parameters relative to the outcome of our conservative approach and no consequences for the overall conclusions were identified (Online Resource, Table A1), supporting our heuristic reconstruction and analyses. Any recent fish extinctions can also be ruled out because brown trout, arctic charr, and three-spined sticklebacks are the only possible fish species for Takvatn due to distributional restrictions from the postglacial period. Nonetheless, our approach can only investigate how the fish introductions appear to have facilitated the colonisation of new consumers to the system, and cannot evaluate potential extinctions associated with these introductions. Extinctions associated with these introductions are possible and could alter our interpretations.

### Food-web analyses and metrics

Topological predator–prey food webs consist of an *n* × *n* matrix of *n* species, with predators as columns and prey as rows (Cohen 1978). Parasites are included by inserting them as columns with their hosts as rows (Lafferty et al. 2006). Binary entries (e.g. 0 or 1) are furthermore inserted in the matrix to indicate consumer-resource links, providing the necessary information to calculate relevant food-web metrics. To explore food-web properties and assess the impact of species additions on food-web structure, we calculated and compared several metrics of the pre- and post-introduction food webs, including species richness (*S*), number of links (*L*), linkage density (*L*/*S*), connectance (*L*/*S* ^2^), and relative nestedness (see below). To help identify the source of any changes, we used the method advocated by Lafferty et al. (2006) to consider quadrants (subwebs) of a matrix organised by grouping free-living species into the first part of the species list and parasite species into the second part of the list. This allowed us to calculate metrics (1) just for free-living species (i.e. the predator–prey web), (2) for parasites and free-living species but excluding predator–parasite and parasite–parasite links, and (3) for parasites and free-living species including predator–parasite and parasite–parasite links (i.e. the total web including parasites). To estimate nestedness, we first calculated matrix temperature (a measure of absolute nestedness) using software described by Rodríguez-Gironés and Santamaría (2006). To allow easier comparison among networks, we then estimated relative nestedness after Bascompte et al. (2003). We also conducted robustness analyses of the before and after webs. Prior to robustness analysis, predator–parasite links were removed, as per Lafferty and Kuris (2009), which also contains details on the analyses. In short, species were removed in a random sequence. At each removal, species lacking resources (or for which any stage has no resources) were also removed. This is a very conservative way to measure interactions among species as it does not account for how consumers affect prey nor consider interaction strengths or species abundances (Dunne et al. 2002). Robustness, here, is the number of removals needed to reduce the number of species by half (Dunne et al. 2002). We took the modal robustness from 500 disassemblies and also recorded the first and third quartiles.

## Results

**Fig. 1:**
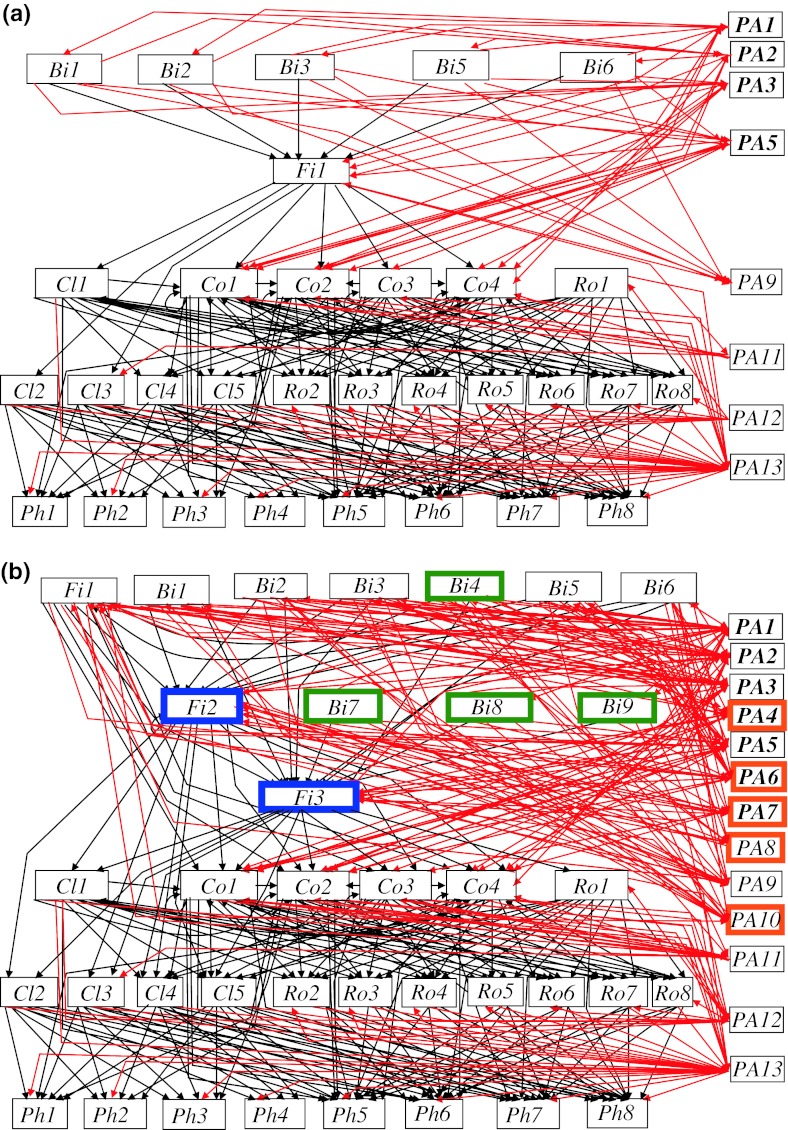
Pelagic food web, including parasites, of the subarctic Lake Takvatn **a** before and **b** after the introductions of arctic charr (*Salvelinus alpinus*) and three-spined stickleback (*Gasterosteus aculeatus*). The two introduced fish species are indicated by *blue boxes*, and the additional bird and parasite species in *green* and *orange boxes*, respectively. Predator–prey links are indicated by *black lines* whereas parasite–host and predator–parasite links are indicated by *red lines*. Nodes include: Phytoplankton: *Asterionella formosa Ph1*, *Cyclotella comensis Ph2*, *Cyclotella kützingiana Ph3*, *Stephanodiscus medius Ph4*, Chrysomona *Ph5*, *Ceratium hirundinella Ph6*, *Gymnodinium helveticum Ph7*, *Elakatothrix genevensis Ph8*. Rotifers: *Asplanchna priodonta Ro1*, *Keratella cochlearis Ro2*, *Kellicottia longispina Ro3*, *Polyarthra* sp*. Ro4*, *Synchaeta* sp*. Ro5*, *Ascomorpha* sp*. Ro6*, *Conochilus unicornis Ro7*, *Filinia gr. longiseta*-*terminalis Ro8*. Cladocerans: *Polyphemus pediculus Cl1*, *Holopedium gibberum Cl2*, *Daphnia galeata Cl3*, *Bosmina longispina Cl4*, *Bosmina longirostris Cl5*. Copepods: *Cyclops scutifer Co1*, *Eudiaptomus graciloides Co2*, *Acanthocyclops gigas Co3*, *Heterocope appendiculata Co4*. Fishes: brown trout *Salmo trutta Fi1*, arctic charr *S. alpinus Fi2*, three-spined stickleback *G. aculeatus Fi3*). Birds: common gull *Larus canus Bi1*, arctic tern *Sterna paradisaea Bi2*, red-breasted merganser *Mergus serrator Bi3*, long-tailed duck *Clangula hyemalis Bi4*, arctic loon *Gavia arctica Bi5*, red-throated loon *Gavia stellata Bi6*, common scoter *Melanitta nigra Bi7*, tufted duck *Aythya fuligula Bi8*, goldeneye *Bucephala clangula Bi9*. Parasites: *Diphyllobothrium dendriticum PA1*, *Diphyllobothrium ditremum PA2*, *Eubothrium crassum PA3*, *Eubothrium salvelini PA4*, *Proteocephalus* sp*. PA5*, *Schistocephalus solidus PA6*, *Philonema oncorhynchi PA7*, *Salmincola edwardsii PA8*, *Gyrodactylus arcuatus PA10*, *Saprolegnia* *PA9*, fungi on crustacean zooplankton *PA11*, *Rotiferophthora* *PA12*, Chytridiomycetes *PA13*. Trophically transmitted parasite species are indicated in *bold*

The reconstructed pre-introduction pelagic food web in Takvatn had 39 nodes, consisting of 8 basal species, 23 free-living predators, and 8 parasitic taxa (Fig. 1a). The basal species were all phytoplankton (*Ph1*–*Ph8*). The predators distributed themselves over four trophic levels (throughout we consider maximum trophic level). The first and second consumer levels comprised rotifers (*Ro1*–*Ro8*) and crustacean zooplankton (*Cl1*–*Cl5* and *Co1*–*Co4*), whereas the third level included a single fish species (brown trout, *Fi1*) and the fourth and top level comprised five bird species (*Bi1*, *Bi2*, *Bi3*, *Bi5* and *Bi6*). The eight parasite taxa present (*PA1*, *PA2*, *PA3*, *PA5*, *PA9*, *PA11*, *PA12* and *PA13*) infected hosts at all trophic levels. 50 % of the parasite species were food transmitted, having hosts at two or more trophic levels.

Comparing the reconstructed pre-introduction web with the current web revealed that the introductions of arctic charr (*Fi2*) and three-spined stickleback (*Fi3*) to the lake resulted in significant changes in the structure of the pelagic food web, including increases in the numbers of nodes and links, as well as an increase in food chain lengths and the number of trophic levels (Fig. 1b; Table 1a). More specifically, the introduction of arctic charr facilitated the arrival of three new parasite species [*Eubothrium salvelini* (*PA4*), *Philonema oncorhynchi* (*PA7*) and *Salmincola edwardsii* (*PA8*)], which all rely upon the charr as their obligate host in this system and are not able to complete their life cycle in the sole presence of brown trout. Similarly, the introduction of three-spined stickleback resulted in the arrival of another two parasite species [*Schistocephalus solidus* (*PA6*) and *Gyrodactylus arcuatus* (*PA10*)] that both have the stickleback as an obligate host in their life cycle. Several of these parasite species also infect many native species in the web during the completion of their life cycles. The fish introductions furthermore opened the possibility for four new bird species [*Clangula hyemalis* (*Bi4*), *Melanitta nigra* (*Bi7*), *Aythya fuligula* (*Bi8*), and *Bucephala clangula* (*Bi9*)] to feed in the pelagic zone. These birds prey on stickleback and arctic charr, but are not able to use brown trout or zooplankton as prey and were thus not integrated in the pre-introduction pelagic web. They may have been foraging along the margins of the lake prior to the fish introductions as they can feed on some benthic invertebrates. However, these four bird species are new for the pelagic network as no suitable prey species were present in the pelagic community prior to the introductions of arctic charr and stickleback. In sum, after the introductions of the two fish species, the number of free-living predators in the pelagic food web increased from 23 to 29, and the number of parasitic taxa from 8 to 13. Furthermore, the number of trophic levels increased from 5 to 6 (Fig. 1; Table 1a).

**Table 1:** Summary of food-web metrics in the pre- and post-introduction pelagic web of the subarctic lake Takvatn including (a) the total web including parasites, (b) the predator–prey subweb, (c) the parasite–host subweb, and (d) the predator–parasite subweb

Parameters	(a) Total web		(b) Predator–prey subweb		(c) Parasite–host subweb		(d) Predator–parasite subweb	
	Pre-introd. web	Post-introd web	Pre-introd. web	Post-introd web	Pre-introd web	Post-introd. web	Pre-introd. web	Post-introd. web
No. of consumer levels	4	5	4	5	–	–	–	–
No. of species	39	50	31	37	39	50	39	50
Potential no. of links	1,521	2,500	961	1,369	248	481	248	481
Observed no. of links	282	440	165	198	43	75	74	167
Linkage density	7.23	8.80	5.32	5.35	1.10	1.50	1.90	3.34
Connectance	0.185	0.176	0.172	0.145	0.173	0.156	0.298	0.347
Omnivory^a^	1.58	2.05	1.43	1.86	1.88	2.23	–	–
Relative nestedness	0.16	0.25	0.09	0.10	–	–	–	–

^a^ *Omnivory* average no. of trophic levels being fed on

The species composition and structure at the basal and first and second consumer levels were apparently unchanged from the pre- to the post-introduction pelagic food web, which may be a result of the conservative approach that was used for the reconstruction of the pre-introduction web. Large changes were, in contrast, evident for the higher trophic levels (birds, brown trout, parasites) following the introductions of charr and stickleback. At the third consumer level, the introduced three-spined stickleback became the principal consumer of zooplankton (Fig. 1). The introduced arctic charr were positioned at the fourth consumer level, preying on stickleback as well as zooplankton. The brown trout prey on the introduced stickleback and arctic charr, and have thus advanced two trophic levels up to the fifth consumer level (Fig. 1). This level also included six species of piscivorous birds (*Bi1*–*Bi6*), which consume both arctic charr and stickleback, whereas three bird species (*Bi7*–*Bi9*) that are only able to use sticklebacks as fish prey were located at the fourth trophic level. Both the pre- and post-introduction food webs were “wasp-waisted” (Fig. 1), with high diversity in the low and high trophic levels and an intermediate level represented only by a single species (brown trout or three-spined stickleback, respectively).

**Fig. 2:**
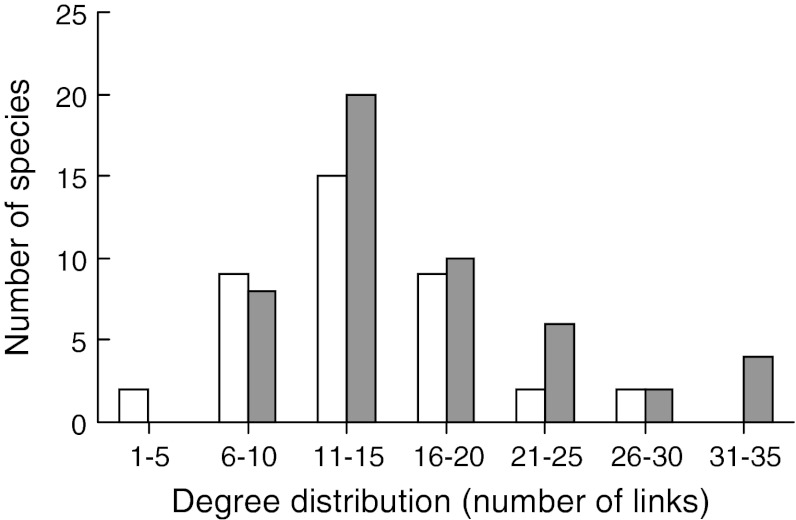
Frequency distributions of links in the Takvatn pelagic food web before (*open bars*) and after (*shaded bars*) the fish introductions; parasites are included

**Fig. 3:**
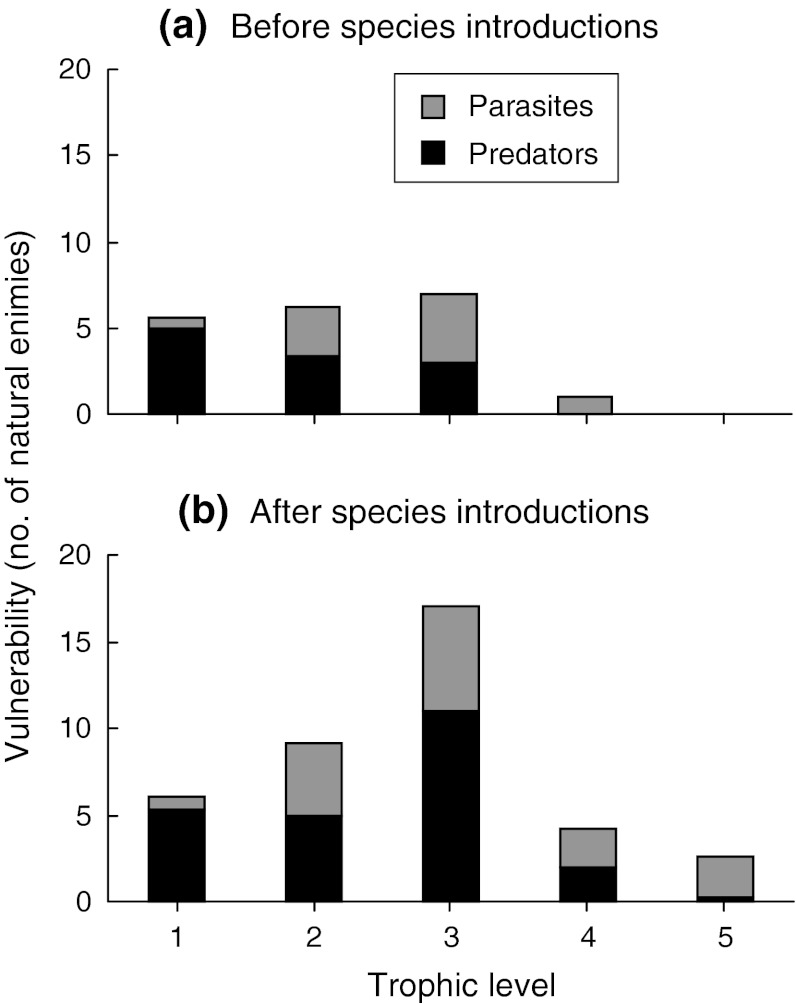
Vulnerability to natural enemies (a statistic of topological food webs representing the number of natural enemy species that feed on a particular species) at different trophic levels of the Takvatn pelagic food web **a** before and **b** after the fish introductions

The fish introductions increased several aspects of system complexity. The reconstructed pre-introduction pelagic food web had 282 links, while the post-introduction web had 440 (Table 1a). The linkage density increased from 7.23 to 8.80, largely as a result of increased predator–parasite links. Connectance decreased (0.185 vs. 0.176) from the pre- to the post-introduction web (Table 1a). The degrees of a node (i.e. the number of links a species has to other species) increased from the pre-introduction (mean 13.4 ± 0.94 SE) to the post-introduction (mean 16.1 ± 0.99 SE) food-web (Fig. 2). Also, the mean degree of omnivory (i.e. the average number of trophic levels being fed on) increased from 1.58 (±0.17) to 2.05 (±0.16) from the pre- to the post-introduction web. Similarly, the number of natural enemies per consumer (i.e. the vulnerability of the species) showed a considerable increase from the pre- to the post-introduction web, particularly at the third and higher trophic levels (Fig. 3). Both the pre- and post-introduction food webs were nested in comparison to randomised matrices (Monte Carlo re-sampling, *P* < 0.0001). The relative nestedness increased from 0.16 to 0.25 from the pre- to the post-introduction web. High nestedness indicates that specialists are more likely to have a diet that is a subset of generalists than to have unique diets.

Parasites drove many of the observed changes in the food web as 79 % of the 158 new-established links after the species additions involved parasites. In particular, the importance of predator–parasite links increased after the species introductions (Table 1d), whereas only moderate differences in network metrics were seen between the pre- and post-introduction parasite–host subwebs (Table 1c). For the free-living predator–prey subweb (i.e. without the parasites included), there were also few differences in network metrics between the pre- and post-introduction networks (Table 1b). For instance, both linkage density and nestedness showed insignificant changes from the pre- to the post-introduction situation of the predator–prey subweb, whereas distinct changes were seen for the total web including parasites. A notable exception is connectance, which showed a larger decrease in the predator–prey web (−16 %) relative to the total web (−4 %). For the total web, robustness decreased from 45 % (quartile range 41–47 %) in the pre-introduction to 43 % (39–46 %) in the post-introduction network. In contrast, if parasites were excluded, robustness increased from 46 % (43–48 %) in the pre-introduction to 47 % (4–48 %) in the post-introduction web.

The body sizes of the free-living species in the pelagic food web increased with increasing trophic level, ranging over several orders of magnitude from basal phytoplankton at 10 μm to top predators at >50 cm in body length (Table 2). In general, there was an increase in body length of about one order of magnitude between consecutive trophic levels. However, in the pre-introduction web, the centimetre size class (10^−2^ m) was missing due to the absence of three-spined stickleback and arctic charr and the fact that native brown trout use tributary streams as nursery areas and only enter the lake at sizes >10 cm (Persson et al. 2007).

**Table 2:** Body size (length ranges) of common free-living species in the Takvatn pelagic food web and their trophic level in the pre- and post-introduction webs

Taxon	Species	Body size	Trophic level	
			Pre-introd. web	Post-introd. web
Phytoplankton^a^	*Asterionella formosa*	40–130 μm	0	0
	*Cyclotella* spp.	10–40 μm	0	0
	Chrysomonads	30–70 μm	0	0
	*Ceratium hirundinella*	200–300 μm	0	0
	*Gymnodinium helveticum*	20–30 μm	0	0
Rotifers^a^	*Keratella cochlearis*	80–320 μm	1	1
	*Kellicottia longispina*	450–860 μm	1	1
	*Conochilus unicornis*	200–380 μm	1	1
	*Asplanchna priodonta*	420–1,500 μm	2	2
Cladocerans^b^	*Daphnia galeata*	0.6–2 mm	1	1
	*Bosmina* spp.	0.2–0.9 mm	1	1
Copepods^b^	*Cyclops scutifer*	0.4–1.5 mm	2	2
	*Eudiaptomus graciloides*	0.4–1.3 mm	2	2
Fish^b^	Three-spined stickleback	1–7 cm	Absent	3
	Arctic charr	5–45 cm	Absent	4
	Brown trout	11–60 cm	3	5
Birds^c^	Ducks	50–54 cm	Absent	4
	Gulls and terns	33–45 cm	4	5
	Mergansers and loons	60–73 cm	4	5

^a^Streble and Krauter (2006) (literature-retrieved data)

^b^Own observations

^c^Mullarney et al. (2000) (literature-retrieved data)

## Discussion

The introduction of arctic charr and three-spined stickleback altered the Takvatn pelagic food web, increasing species richness beyond the mere addition of the two introduced species by facilitating the establishment of several hitchhiking or independently arriving parasite species and constituting essential food resources for new avian predators. Hence, the introductions of the 2 fish species facilitated the addition of another 9 species to the pelagic community, increasing richness from 39 to 50 species. These species additions also resulted in a large increase in the number of trophic links, and the topology of the food web changed dramatically. The increases in numbers of nodes and links were accompanied by an increase in food-chain lengths and in the total number of trophic levels of the web. Moreover, the complexity of the web increased, clearly manifested by increases in linkage density, degree distribution, vulnerability to natural enemies, omnivory, and nestedness, which potentially also has large consequences for network functioning and stability (e.g. Allesina and Pascual 2008; Lafferty et al. 2008). The connectance changed little in the total pelagic web, but decreased in the predator–prey web. Other important food-web characteristics such as linkage density and nestedness exhibited few changes from the pre- to the post-introduction predator–prey web. Jonsson et al. (2005) similarly found modest effects on the pelagic predator–prey web of Tuesday Lake following a food-web manipulation including both addition and removal of fish species. Parasites were not included in the Tuesday Lake studies, but Jonsson et al. acknowledged their potential importance and also called for an exploration of the role of parasites in food-web manipulations such as species additions (Jonsson et al. 2005). Our study indeed confirms that parasites can have an important role in food-web alterations following species introductions. More specifically, the large differences observed in responses between the predator–prey and the total web were due to the fact that most of the new links were parasite-associated; an observation that underpins the importance of taking parasites into considerations in food-web studies (see also, e.g., Marcogliese 2003, 2005; Lafferty et al. 2008; Beckerman and Petchey 2009; Poulin 2010).

Due to the large modifications in species composition following the introductions of the two fish species to Takvatn, there were also large alterations in functional traits within the food web related to changes in size–structure interactions and foraging efficiencies within the network; impacts that also influence parasite transmission within the system (see also Poulin and Leung 2011). At 10^−2^ m in length, stickleback and juvenile arctic charr became a new intermediate size class in the pelagia of Takvatn (see Table 2), enhancing the size coherence within the pelagic web and resulting in (1) increased predation on crustacean zooplankton (see below), (2) a two-step advancement in trophic position of brown trout, and (3) the additional invasion of the pelagic web by bird species feeding on stickleback and juvenile charr. Brown trout, the only fish species present in the pre-introduction web, make limited use of zooplankton as prey, and arctic charr and stickleback are known to be far more efficient zooplankton predators (Langeland and Nøst 1995). Hence, the pre-introduction crustacean zooplankton community in Takvatn must have experienced much less predation from brown trout alone than in the subsequent presence of the introduced arctic charr and three-spined stickleback. While it is possible that the introduced fish drove some large zooplankton extinct, it is hard to reconstruct pre-invasion food webs. We found that an inclusion of the most likely extinction (*Bythotrephes longimanus*) in the reconstructed pre-invasion web did not alter the general findings (see Online Resource). However, whether or not extinctions occurred, we do not wish to understate the importance of fish predation on this system. Increased fish predation on crustacean zooplankton changes the zooplankton community towards more mobile species like copepods and towards smaller species, in particular within the cladocerans (e.g. Gliwicz and Pijanowska 1989). Accordingly, the post-introduction crustacean zooplankton community in Takvatn has been dominated by small-sized cladocerans and copepods (Dahl-Hansen 1995; Primicerio 2000), which have also become important prey of planktivorous arctic charr and three-spined stickleback (Amundsen and Klemetsen 1988; Jørgensen and Klemetsen 1995). Several fish parasites (including *Diphyllobothrium* spp., *Eubothrium* spp., *S. solidus*, *P. oncorhynchi*, and *Proteocephalus* sp.) use copepods as intermediate hosts, and predation-induced changes in the zooplankton community have thus likely enhanced the completion of the life cycles of these parasites (Knudsen 1995). Intensified piscivory by birds related to the presence of three-spined stickleback and arctic charr has furthermore increased the transmission rates of several bird parasites that use fish as intermediate hosts (Knudsen et al. 1996). These trophically transmitted parasites are highly connected species that are present at several trophic levels (Amundsen et al. 2009), and they contribute to the complexity of the network, thus highlighting the particular importance of food-transmitted parasites in food webs (see also Lafferty et al. 2006, 2008; Hernandez and Sukhedo 2008; Poulin and Leung 2011).

The introduction of three-spined stickleback has likely increased the transmission rate and abundance of food-transmitted parasites in both piscivorous birds and fish in the system as this small-sized fish is a common prey of these predators (e.g. Whoriskey and Fitzgerald 1985; L’Abée-Lund et al. 1992; Amundsen 1994) and also acts as an intermediate host for several of the present parasite species (Amundsen et al. 2009). This is true for the two *Diphyllobothrium* species that are transmitted as plerocercoid larvae from sticklebacks as well as small- and intermediate-sized arctic charr and brown trout to their final bird hosts (Vik 1957; Halvorsen 1970). The plerocercoid larvae can also use large piscivorous individuals of brown trout and arctic charr as paratenic hosts (Halvorsen and Wissler 1973). Furthermore, predation rates may increase along with transmission due to parasite-induced host behavioural modifications that make infected individuals more vulnerable to predation (Moore 2002). In Takvatn, up to 60 % of sticklebacks older than 1 year may carry larvae of the tapeworm *S. solidus* during summer and early autumn (P.-A. Amundsen et al., unpublished data). Infections with *S. solidus* render stickleback more vulnerable to predation both from piscivorous birds and fish due to behavioural manipulation of the hosts (e.g. Barber et al. 2004). This host–behaviour manipulation enhances the transmission of *S. solidus* to their final hosts, but will also facilitate the transmission of other larval parasites infecting stickleback, emphasising the strong and complex food-web interrelationships between predation and parasitism.

Our approach of reconstructing the food web to estimate conditions prior to invasion was dictated by a lack of records about the historical food web. Ideally, we would have also been able to study replicated scenarios, but this option was not available to us. Future studies could compare the food webs of lakes with and without introduced fishes. Our results provide a starting point for such an effort. Similarly, we limited ourselves to the pelagic component of the food web because this was the part of the system where we had sufficient data. Our view of the system could change if we were able to add benthic species to the web. This will be an area of future work. We have more detailed ecological data on some of the species and their feeding interactions, but the only way for us to capture the complexity of the entire system was to use the topological abstraction. Although topological webs like ours provide a simple description of complexity and are amenable to descriptive statistics, they sacrifice important aspects of how energy flows through the system.

In conclusion, our study reveals large food-web alterations in the pelagic community following the introduction of arctic charr and three-spined stickleback into Lake Takvatn. Both species have become engaged in many trophic links and constitute important hubs in the post-introduction network. The introduction of these two fishes also facilitated several additional species entering the pelagic network, including new parasite species and avian predators, leading to large changes in species richness and food-web structure. Our study also reveals several associated consequences for the functioning of the pelagic web with potential implications for robustness, resilience and stability of the ecological community. Parasites, in particular trophically transmitted species, have a prominent role in the structure and function of this food web. Most notably, network topology and trophic dynamics can be altered after the addition of new free-living species and their associated parasites.

## Electronic supplementary material

Below is the link to the electronic supplementary material.

Supplementary material 1 (DOC 66 kb)
