# Supplementary material for: New parasites and predators follow the introduction of two fish species to a subarctic lake: implications for food-web structure and functioning
Source: Oecologia. 2012 Sep 28;171(4):993–1002. doi: 10.1007/s00442-012-2461-2 (PMC3612402; doi:10.1007/s00442-012-2461-2)
Supplement: Supplementary file 1 — Supplementary material 1 (DOC 66 kb) [file 442_2012_2461_MOESM1_ESM.doc]

**ELECTRONIC SUPPLEMENTAL MATERIAL**

**New parasites and predators follow the introduction of two fish species to a subarctic lake: implications for food-web structure and functioning**

Per-Arne Amundsen • Kevin D. Lafferty • Rune Knudsen • Raul Primicerio • Roar Kristoffersen • Anders Klemetsen • Armand M. Kuris

Appendix 1: **Analysis of alternative reconstructions of the pre-introduction web**

Our heuristic reconstruction of the pre-introduction network follows a conservative approach, only omitting species that rely upon Arctic charr and/or three-spined stickleback as obligate nutritional resources. Alternative reconstructions of the pre-introduction web are however possible. In particular, top-down or cascading predation effects may have occurred following the fish introductions with potential consequences for the species composition at lower trophic levels, although the available species pool in this subarctic region is restricted (Aagaard and Dolmen 1996). The most plausible alternative scenarios are as follow:

a) The large-bodied clodoceran *Bythotrephes longimanus* occurs in nearby lakes and could potentially have been extirpated from Takvatn after the arrival of Arctic charr and stickleback. This predation-vulnerable cladoceran species was also recently (August 2010) observed for the first time in zooplankton samples from Takvatn, likely as a result of an extensive fish stock manipulation in the lake in the 1980’s (Amundsen et al. 1993) after which the population density of piscivorous brown trout have increased (Persson et al. 2007), whereas zooplankton-feeding Arctic charr and three-spined stickleback have declined (Klemetsen et al. 2002, Person et al. 2007, P.-A. Amundsen et al., unpublished).

b) The small-sized *Bosmina longirostris* has low competitive ability against larger cladocerans, and its presence is therefore most common under heavy size-selective predation pressure from planktivorous fish (Dahl-Hansen et al. 1994, Perga et al. 2010). Hence, the presence of *B. longirostris* in the post-introduction web may be facilitated by the presence of three-spined stickleback as a keystone predator, and this cladoceran may thus have been absent in the pre-introduction web.

c) The phytoplankton composition of the pre-introduction web may have differed from the post-introduction web related to trophic cascade effects from possible changes in the crustacean zooplankton community. The presence of a large-sized cladoceran like *B. longimanus* could potentially both decrease and increase the diversity of phytoplankton, depending on the interactions involved.

We implemented these different scenarios in alternative reconstructions of the pre-invasion web and re-estimated important food-web parameters. This analysis revealed only minor differences in these parameters relative to the outcome of our conservative approach and no change in the overall conclusions (Table A1), demonstrating that our heuristic reconstruction approach is robust to the most likely extinction scenario and other plausible changes in species composition. However, as noted in the text, even if the introductions did not result in significant extinctions, we do expect that they greatly decreased the relative abundance of large-bodied zooplankton, with numerous indirect effects on the food web (i.e., not only those detectable through an analysis of topology).

The topological approach to analysing ecological communities is widely used because it requires few assumptions about the way that species interact. For instance, topological webs do not usually include interaction strengths among species, and this makes them relatively easy to construct. A food-web topology is an ecological map of who eats whom in a defined ecological system. Although networks can be complicated, this complexity can be captured by a variety of simple metrics. Theoretical models have linked topological metrics with system dynamics such as stability. Nonetheless, topological webs are simple, and therefore not intended to provide a complete description of an ecological community or its dynamics.

**References**

Aagaard A, Dolmen D (eds) (1996) Limnofauna Norvegica. Tapir, Trondheim

Amundsen P-A, Klemetsen A, Grotnes P-E (1993) Rehabilitation of a stunted population of Arctic charr by intensive fishing. N Am J Fish Mgmt 13:483-491

Dahl-Hansen GAP, Rubach S, Klemetsen A (1994) Selective predation by pelagic Arctic charr on crustacean plankton in Takvatn, northern Norway, before and after mass removal of Arctic charr. Trans Am Fish Soc 123:385–394 doi: 10.1577/1548-8659(1994) 123<0385:SPBPAC>2.3.CO;2

Klemetsen A, Amundsen P-A, Grotnes P-E, Knudsen R, Kristoffersen R, Svenning M-A (2002) Takvatn through 20 years: Long-term effects of an experimental mass removal of Arctic charr *Salvelinus alpinus* from a subarctic lake. Env Biol Fish 64:39-47 doi: 10.1023/A:1016062421601

Perga ME, Desmet M, Enters D, Reyss J-L (2010) A century of bottom-up- and top-down-driven changes on a lake planktonic food web: A paleoecological and paleoisotopic study of Lake Annecy, France. Limnol Oceanogr 55:803-816 doi: 10.4319/lo.2009.55.2.0803

Persson L, Amundsen P-A, De Roos AM, Klemetsen A, Knudsen R, Primicerio R (2007) Culling prey promotes predator recovery – alternative states in a whole-lake experiment. Science 316:1743-1746 doi: 10.1126/science.1141412

Table A1. Comparison of the adopted reconstruction approach for the pre-introduction web with plausible alternatives for the network reconstruction. Alt. 1: *Bythotrephes longimanus* included; Alt. 2: *Bosmina longirostris* omitted; Alt. 3: Alt. 1 and 2 combined; Alt. 4: Alt. 3 *plus* one phytoplankton species omitted; Alt. 5: Alt. 3 *plus* one new phytoplankton species added; Alt. 6: Alt. 3 *plus* two phytoplankton species omitted; Alt. 7: Alt. 3 *plus* two new phytoplankton species added.

|  | **Post-intro-duction**  **web** | **Pre-introduction web reconstructions** | | | | | | | |
| --- | --- | --- | --- | --- | --- | --- | --- | --- | --- |
| **Parameters:** |  | Adopted approach | Alt. 1 | Alt. 2 | Alt. 3 | Alt. 4 | Alt. 5 | Alt. 6 | Alt. 7 |
| **a) Total web** |  |  |  |  |  |  |  |  |  |
| **No. of nodes, S** | **50** | **39** | 40 | 38 | 39 | 38 | 40 | 37 | 41 |
| **No. of links, L** | **440** | **282** | 298 | 267 | 298 | 269 | 289 | 255 | 303 |
| **Linkage density** | **8.80** | **7.23** | 7.45 | 7.03 | 7.64 | 7.08 | 7.23 | 6.89 | 7.39 |
| **Connectance** | **0.176** | **0.185** | 0.186 | 0.185 | 0.196 | 0.186 | 0.181 | 0.186 | 0.180 |
| **Omnivory^1^** | **2.05** | **1.58** | 1.68 | 1.55 | 1.65 | 1.65 | 1.65 | 1.65 | 1.65 |
|  |  |  |  |  |  |  |  |  |  |
| **b) Predator-prey web** |  |  |  |  |  |  |  |  |  |
| **No. of nodes, S** | **37** | **31** | 32 | 30 | 31 | 30 | 32 | 29 | 33 |
| **No. of links, L** | **198** | **165** | 178 | 151 | 178 | 151 | 169 | 138 | 182 |
| **Linkage density** | **5.35** | **5.32** | 5.56 | 5.03 | 5.74 | 5.03 | 5.28 | 4.76 | 5.52 |
| **Connectance** | **0.145** | **0.172** | 0.174 | 0.168 | 0.185 | 0.168 | 0.165 | 0.164 | 0.167 |
| **Omnivory^1^** | **1.86** | **1.43** | 1.57 | 1.39 | 1.52 | 1.52 | 1.52 | 1.52 | 1.52 |

^1)^ Omnivory = average no. of trophic levels being fed on
